# Impact of a reduced iodine load with deep learning reconstruction on abdominal MDCT

**DOI:** 10.1097/MD.0000000000034579

**Published:** 2023-09-01

**Authors:** Gaspard Ludes, Mickael Ohana, Aissam Labani, Nicolas Meyer, Sébastien Moliére, Catherine Roy

**Affiliations:** a Department of Radiology B, University Hospital of Strasbourg – New Civil Hospital, Strasbourg, Cedex, France; b Department of Statistics, University Hospital of Strasbourg – New Civil Hospital, Strasbourg, Cedex, France.

**Keywords:** adaptive iterative reconstruction, computed tomography, contrast media, deep learning-based image reconstruction, radiation dose

## Abstract

To evaluate the impact of a reduced iodine load using deep learning reconstruction (DLR) on the hepatic parenchyma compared to conventional iterative reconstruction (hybrid IR) and its consequence on the radiation dose and image quality. This retrospective monocentric intraindividual comparison study included 66 patients explored at the portal phase using different multidetector computed tomography parameters: Group A, hybrid IR algorithm (hybrid IR) and a nonionic low-osmolality contrast agent (350 mgI/mL); Group B, DLR algorithm (DLR) and a nonionic iso-osmolality contrast agent (270 mgI/mL). We recorded the attenuation of the liver parenchyma, image quality, and radiation dose parameters. The mean hounsfield units (HU) value of the liver parenchyma was significantly lower in group B, at 105.9 ± 10.9 HU versus 118.5 ± 14.6 HU in group A. However, the 90%IC of mean liver attenuation in the group B (DLR) was between 100.8 HU and 109.3 HU. The signal-to-noise ratio of the liver parenchyma was significantly higher on DLR images, increasing by 56%. However, for both the contrast-to-noise ratio (CNR) and CNR liver/PV no statistical difference was found, even if the CNR liver/PV ratio was slightly higher for group A. The mean dose-length product and computed tomography dose index volume values were significantly lower with DLR, corresponding to a radiation dose reduction of 36% for the DLR. Using a DLR algorithm for abdominal multidetector computed tomography with a low iodine load can provide sufficient enhancement of the liver parenchyma up to 100 HU in addition to the advantages of a higher image quality, a better signal-to-noise ratio and a lower radiation dose.

## 1. Introduction

In recent decades, the use of abdominal multidetector computed tomography (MDCT) has significantly increased owing to its excellent diagnostic performance, easy accessibility, short scanning time, and cost-effectiveness. However, the widespread use of computed tomography (CT) has led to increased radiation exposure.^[1]^ To address this concern, various dose optimization techniques have been developed that substantially reduce the overall radiation dose.^[2]^ However, reducing the radiation dose can amplify the noise in CT images or create a “plastic-like” appearance that may degrade the diagnostic value of the examination.^[3]^

Recently, deep learning reconstruction (DLR) techniques have been developed that incorporate convolutional neural networks into the reconstruction process to remove image noise (N) and improve the spatial resolution. Some studies have suggested that the DLR algorithm increases the signal-to-noise ratio (SNR), contrast-to-noise ratio (CNR), and overall image quality compared to previous algorithms. This novel reconstruction approach is also expected to provide substantial dose reduction while maintaining diagnostic image quality.^[3–5]^ Therefore, implementation of this technique is promoted as a major adjunct to successful diagnostic CT.^[6]^

To our knowledge, no study has evaluated the potential efficiency of a lower iodine load using the DLR algorithm. The purpose of this study was to evaluate the impact of a reduced iodine load using the DLR technique on abdominal CT with a focus on the hepatic parenchyma. In this study, we conducted an intra-individual comparison of the DLR technique with conventional iterative reconstruction (hybrid IR) and assessed the impact on radiation dose and image quality.

## 2. Materials and methods

**Figure 1.**
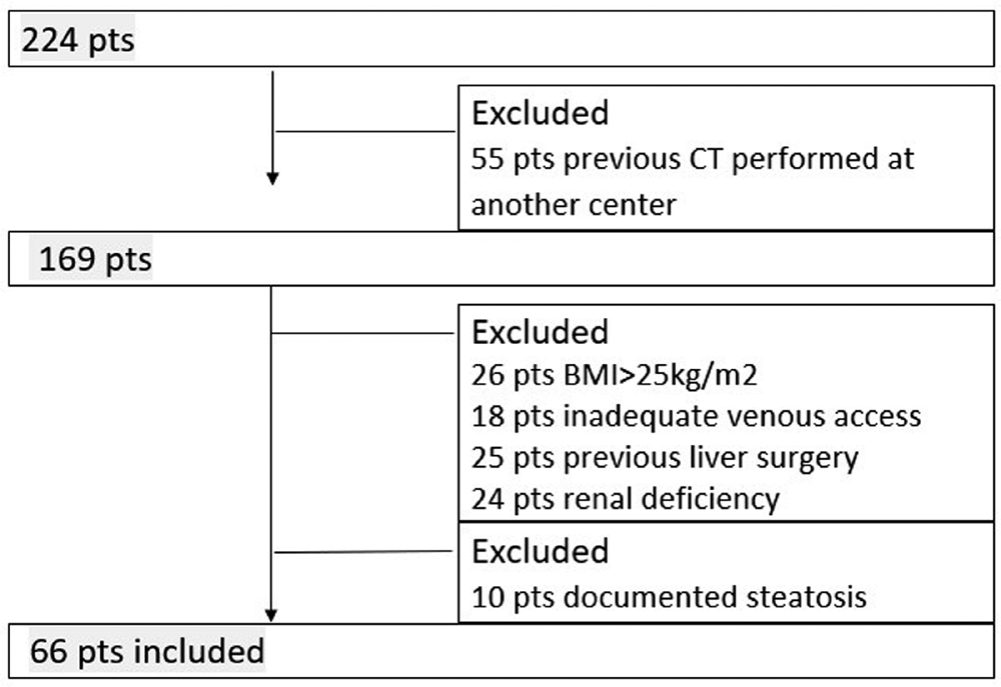
Flow chart shows the study design with exclusion and inclusion criteria.

From January 2021 to January 2022, among a large group of 224 patients explored in the context of routine oncologic follow-up, we excluded 148 patients (55 patients with previous CT performed at another center, 26 cases with a body mass index (BMI) up to 25 kg/m^2^, 18 cases with an inadequate antecubital venous access to obtain a homogeneous injection rate, 25 cases with a history of surgical operation of the liver, and 24 cases with glomerular filtration rate <60 mL/min. Ten cases with documented steatosis were also excluded, as they could vary between successive examinations. A flow chart is shown in Figure 1.

This retrospective study was approved by our institutional review board and written informed patient consent was not required. The patients were orally informed that their second CT examination would be performed using the newest CT machine in our department.

We evaluated 2 successive MDCT explorations with a time interval between 6 and 9 months (mean time interval: 227 ± 45 days) for each patient, using 2 different image reconstruction technologies and 2 types of contrast agents.

The first CT examination (Group A) was performed on an Aquilion One GENESIS Edition (Canon Medical Systems, Otawara, Japan) using a conventional hybrid iterative reconstruction algorithm, adaptive iterative dose reduction 3-dimensional (AIDR 3D), and a nonionic low-osmolality contrast agent with a high iodine concentration of 370 mgI/mL (Ultravist 370, Bayer Schering Pharma, Berlin, Germany). The second CT examination (Group B) was performed on our more recent Aquilion One PRISM Edition (Canon Medical Systems, Otawara, Japan) using a DLR technique, advanced intelligent Clear-IQ engine (AiCE), and a nonionic iso-osmolality contrast agent with a lower iodine load of 270 mgI/mL (Visipaque 270, GE Healthcare, Chicago, IL).

All patients received intravenous 95 ± 1 mL of contrast agent at a constant flow rate of 3.5 mL/s via the antecubital vein, followed by a saline chaser (50 mL of 0.9% sodium chloride solution) administered at the same flow rate. The total iodine load was 35.1 grI (group A) and 25.6 grI (group B). Scanning was performed at a fixed delay of 75 seconds after the initiation of the contrast injection to obtain images in the portal venous phase. Bolus tracking was not performed.

All scan parameters were kept identical between the 2 examinations for each patient, including helical acquisition, scan start and end locations at the top of the shoulder, and under the pubic symphysis. The automatic exposure control (SURE Exposure 3D) was used for all scans and defined as “Standard + 1” for AiCE with a noise index set at 10.0 for SD with a range of 140 to 550 mA versus “Standard” for AIDR 3D with a noise index set at 11.5 for SD with a mA range of 80 to 650 mA. The acquisition time was varied from 10 to 11 seconds (Table 1).

**Table 1:** Multidetector computed tomography (MDCT) acquisition parameters of the 2 groups A and B.

Parameters	Group A (AIDR 3D) IR	Group B (AiCE) DLR
Acquisition mode	Helical	Helical
Tube voltage (kVp)	120	120
Tube current (mA)	80–650	140–550
Collimation (mm)	0.5 × 80 row	0.5 × 80 row
Rotation time (s)	0.5	0.5
Field of view (mm)	380	380
Slice thickness (mm)	1.0	1.0
Interval (mm)	0.8	0.8
Pitch	0.813	0.813
Matrix size	512	512

AiCE = advanced intelligent clear-IQ engine, AIDR 3D = adaptive iterative dose reduction 3-dimensional, DLR = deep learning reconstruction, IR = iterative reconstruction.

The dose-length product (DLP) values and computed tomography dose index volume (CTDIvol) displayed on the CT console were recorded.

### 2.1. Quantitative analysis.

All images were analyzed using the VITREA workstation version 7.11 (Vitrea-Canon Medical Informatics, Minneapolis, Minnesota)

Abdominal CT images were assessed using soft-tissue window settings (window level, 40 hounsfield units [HU]; window width, 400 HU).

The measurements were conducted by a radiologist with 4 years of experience (x). All measurements were performed on a slice that intersected the portal vein. A circular ROI with a fixed area of 1.0 cm^2^ was placed in a homogeneous part of the right and left hepatic lobes, as well as inside the portal vein, in the same location for both examinations. The average CT density in HU, along with the standard deviation of the CT value (SD), was obtained from 3 measurements taken at location close to each other. In addition, we recorded the average CT density in the erector spinae muscles, inside the main portal vein, and inside the extra corporeal air in front of the anterior abdominal wall.

Noise was defined as the mean of the standard deviation (N) of the signal measurement in extracorporeal air.

The SNR and CNR were calculated using the following formulas:

SNR = mean hepatic parenchyma attenuation/standard deviation in extra-corporal air (N)

CNR = (hepatic parenchyma attenuation − erector spinae muscle CT attenuation)/mean standard deviation of erector spinae muscle

CNR liver/PV = attenuation of hepatic parenchyma − attenuation of portal vein)/mean standard deviation of erector spinae muscle

### 2.2. Qualitative analysis

Image quality (IQ) was assessed by 2 radiologists with 4 (x) and 25 years (xx) experience in abdominal imaging and prior experience of reading CT images with DLR. Both radiologists were blinded to patient data and reconstruction algorithm. Both investigators had prior experience of reading CT images with DLR.

Readers were asked to subjectively and independently rate the overall image quality on the 2 sets of MDCT examinations using a 5-point scale ranging from 1 to 5:1 = very poor image quality with no diagnostic information, 2 = low image quality that reduces confidence in making a diagnosis, 3 = moderate image quality sufficient for diagnosis, 4 = good image quality clearly demonstrating anatomical structures, and 5 = excellent image quality enabling excellent differentiation of even small anatomical structures.

Disagreements were resolved by consensus with a third reader (xxx).

### 2.3. Statistical analysis

Statistical analysis was conducted using the XLSTAT statistical software (Addinsoft, Microsoft Excel, version 2019). Interobserver agreement for the qualitative analysis of image quality was assessed using quadratic Cohen weighted kappa statistics (k). Kappa statistics were calculated using 95% confidence intervals. Hereby, kappa values <0 were considered as indicating no agreement, 0.00 to 0.20 as poor, 0.21 to 0.40 as fair, 0.41 to 0.60 as moderate, 0.61 to 0.80 as substantial, and 0.81 to 1.00 as an 19 excellent agreement.

The student paired *t* test was used to evaluate differences between the 2 examinations in terms of attenuation of the liver parenchyma, SNR, CNR, DLP, and CTDIvol. Statistical difference was considered as significant at *P* ≤ .05.

## 3. Results

### 3.1. Patient population

There was no change in the BMI between the 2 examinations. There were no acute or delayed adverse events due to the contrast agent injection, regardless of the group.

A final cohort (Fig. 1) of 66 patients was enrolled in this study, comprising 31 women and 35 men with a mean age of 62 ± 15.7 years old, BMI between 20 and 25 kg/m^2^ and body weight between 70 and 80 kg. The clinical characteristics of the patients included breast cancer (n = 13), gastrointestinal tumors (n = 14), lung cancer (n = 10), and urogenital tumors (n = 29).

### 3.2. Image quality

**Figure 2.**
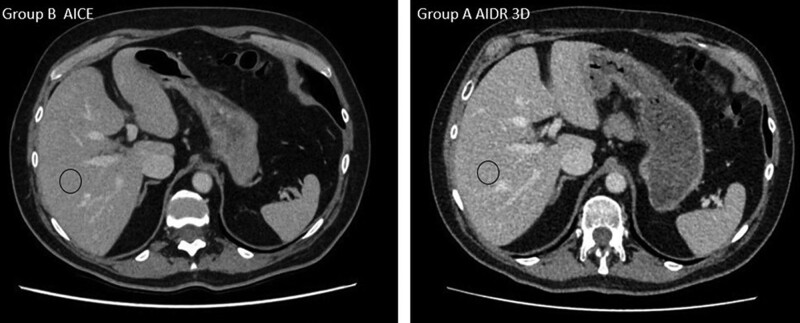
Comparison of the images obtained at the same level in a 65-yr-old woman who underwent a follow-up CT for breast carcinoma. The size and localization of ROI were the same. Note the significantly better image quality achieved with advanced intelligent clear- IQ engine (AiCE). (A) Group A with adaptive iterative dose reduction 3-dimensional (AIDR 3D) (hybrid IR) reconstruction technique − ROI mean value = 115.3 ± 12.3 hounsfield units (HU). Dose-length product (DLP) = 258.4 mGy.cm; signal-to-noise ratio (SNR) = 13.88. (B) Group B with the AICE deep learning reconstruction (DLR) reconstruction technique − ROI mean value = 104.5 ± 9.5 HU. DLP = 353.2 mGy.cm; SNR = 5.85.

The subjective image quality was significantly better with AiCE than with AIDR 3D. Indeed, the mean score (on a Likert scale) was 4.5 ± 0.7 with AiCE versus 3.5 ± 0.9 with AIDR 3D (*P* < .001) (Fig. 2).

No artifacts were present on MDCT images for any of the patients.

The interobserver agreement on image quality was excellent for both groups but was clearly higher in group B (k = 0.92) than in group A (k = 0.83).

The DLR reconstructions AiCE consistently showed a statistically significant decrease in N of 29% compared to the hybrid IR (AIDR 3D) (Table 2).

**Table 2:** Image noise, signal to noise ratio (SNR) and contrast to noise ratio (CNR) of images derived from the 2 reconstruction techniques.

	AIDR 3D (Group A)	AICE (Group B)	*P* value
SNR	5.76 (95% CI: 5.29–6.22)	13.36 (95% CI: 12.68–14.04)	<.0001
CNR	3.46 (95% CI: 3.19–3.65)	3.60 (95% CI: 3.42–3.95)	.895
CNR liver/PV	3.29 (95% CI: 3.11–3.55)	2.96 (95% CI: 2.67–3.24)	.065
N (HU) image noise	17.20 (95% CI: 16.05–18.3)	12.18 (95% CI: 11.2–13.3)	.001

AiCE = advanced intelligent clear-IQ engine, AIDR 3D = adaptive iterative dose reduction 3-dimensional, CNR = contrast-to-noise ratio, HU = hounsfield units, N = image noise, PV = portal vein, SNR = signal-to-noise ratio.

Our findings indicated that the SNR was undeniable higher in DLR images, with a value increased of 56% with a statistically significant difference. However, for both the CNR and CNR liver/PV no statistical difference was found (Table 2), even if the CNR result was slightly higher for group B.

### 3.3. Image analysis

**Figure 3.**
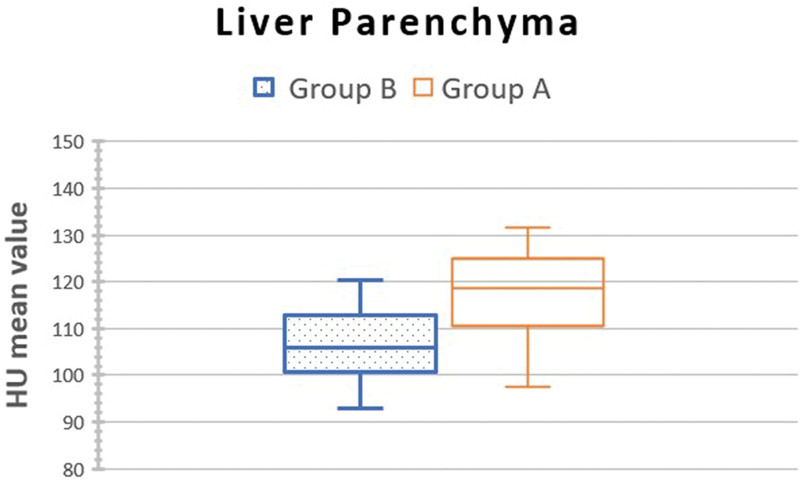
Box plot showing the liver parenchyma mean HU value of the 2 groups. Group A: adaptive iterative dose reduction 3-dimensional (AIDR3D) (hybrid IR). Group B: advanced intelligent clear- IQ engine (AiCE) deep learning reconstruction (DLR).

The mean hepatic parenchymal attenuation was 118.90 ± 14.6 HU (95% CI:110.3–122.2 HU) for group A (AIDR 3D) and 105.9 ± 9.6 HU (95% CI:100.3–109.5 HU) for group B (AICE), *P* = .22 (Fig. 3). There were no statistically significant differences between the 2 groups.

The 90%IC of mean liver attenuation in group B (DLR) was 100.8 to 107.9 HU, meaning the true value is superior to 100.8 HU in 95% of cases.

The main portal vein attenuation was 180 ± 31.1 HU for group A (AIDR 3D) and 145.8 ± 23.2 HU for the group B (AICE) *P* < .001. There was a statistically significant difference between the 2 groups; however, for the CNR liver/PV, there was no significant difference between the 2 groups (*P* = .065).

### 3.4. Radiation dose

**Figure 4.**
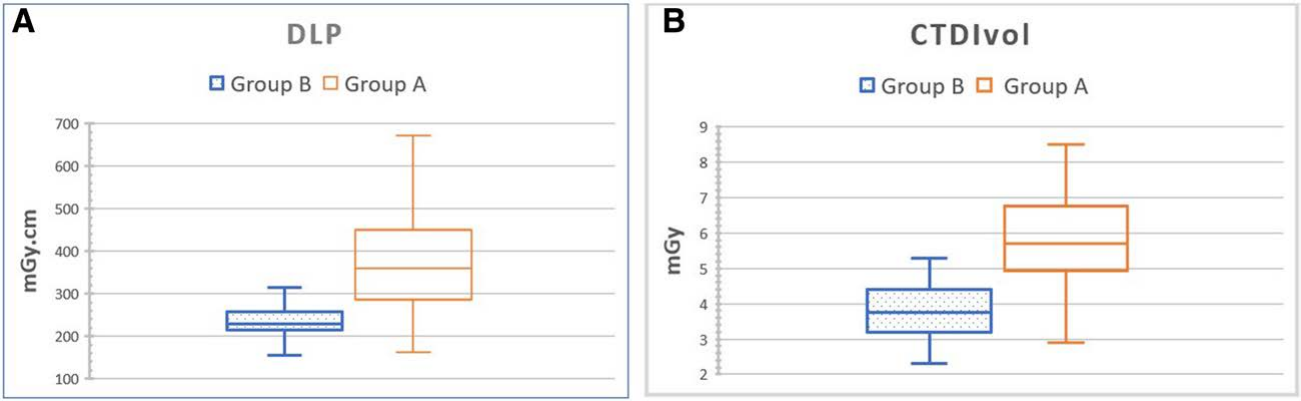
Box plot showing the radiation dose parameters deep learning reconstruction (DLR) and computed tomography dose index volume (CTDIvol). Group A: adaptive iterative dose reduction 3-dimensional (AIDR3D) (hybrid IR). Group B: advanced intelligent clear- IQ engine (AiCE) deep learning reconstruction (DLR).

The mean DLP value was significantly lower with AiCE (238.10 ± 45.39 mGy·cm) than with AIDR 3D (359.6 ± 143.1 mGy·cm) (*P* < .001), corresponding to a significant radiation dose reduction of 36% for DLR (Fig. 4A).

The mean CTDIvol value for hybrid IR (AIDR 3D) and DLR (AiCE) were 5.77 ± 2.06 mGy and 3.75 ± 0.77 mGy, respectively, corresponding to a significant radiation dose reduction of 35% for DLR compared to hybrid-IR. (*P* < .001), respectively (Fig. 4B).

## 4. Discussion

In recent years, a remarkable progress has been made in image reconstruction techniques for CT image acquisition.^[7]^ Filtered back projection (FBP) was the initial standard for CT image reconstruction, which was later combined with iterative reconstruction (IR) and hybrid IR algorithms. Although IR algorithms enable a significant reduction in both radiation dose and N, the resulting image texture appearance can be confusing because of its plastic-like appearance and unnatural aspect for diagnostic purposes. In our study, the DLR algorithm was calculated from a modified IR, so that this surprising image appearance is minimal. Even if, in theory when reducing the dose, there is an impairment of the characteristics of low contrast areas, we did not observe a statistically significant difference in the CNR by using the erector spinae muscle value.

Recently, DLR techniques that incorporate convolutional neural networks into the reconstruction process to remove N and improve spatial resolution have been implemented in CT devices. Currently, 2 vendor-specific DLR algorithms are clinically available. Recent studies on abdominal CT have clearly demonstrated that DLR images show significantly higher SNR and CNR and maintain a higher level of image quality, in addition to a substantial reduction in radiation dose by approximately 50% compared to hybrid IR.^[3,6,8–14]^

Our results are consistent with these findings, indicating that DLR allows for significantly higher image quality, with an improvement in the SNR of 56% and an approximately similar CNR. Our radiation dose reduction with DLR was calculated to be 35% compared to that with hybrid IR, which is lower than that reported in previous studies. This may be due to the threshold of the automatic exposure control that was selected in our study.

In the past, it has been shown that contrast enhancement of abdominal organs is directly affected by the total iodine dose and iodine delivery rate. Several studies have compared organ enhancement on abdominal MDCT, but there is wide heterogeneity among protocols in terms of the concentration of contrast agent, total iodine load, injection rate and CT parameters. Additionally, contrast enhancement depends on multiple individual factors such as BMI, cardiac output and circulation time. Therefore, an intraindividual comparison of contrast enhancement measurements is preferable to an interindividual correlation to avoid potential bias and non-comparability.^[15–18]^

In the last decade, studies have shown that using a low iodine load of contrast agent, such as 270 mgI/mL, and a lower tube voltage (80 kVp) with advanced reconstruction techniques, such as IR, can improve hepatic enhancement while reducing iodine load compared to more conventional contrast agents (350 mgI/mL or 370 mgI/mL), and maintain adequate image quality.^[19–21]^

In a recent study by Choi et al,^[22]^ it was shown that despite a lower SNR and CNR of abdominal organs with 240 mgI/mL contrast agent compared to 320 mgI/mL contrast agent and a similar radiation dose, a concentration of 240 mgI/mL was feasible for evaluating the liver. However, this study was performed using 2 different CT technologies, single and dual energy, using a lower tube voltage.

Previous studies have also shown the feasibility of using a 240 mgI/mL contrast medium in CT angiography and CT urography with a hybrid-IR algorithm.^[23,24]^ In more recent studies comparing DLR to hybrid IR in abdominal MDCT, no significant differences in CT attenuation values were found between the 2 reconstruction algorithms for the liver parenchyma in the portal phase.^[8,13,25–28]^

However, a meta-analysis showed a trend towards higher CT attenuation values for DLR compared to hybrid IR, although this difference was not statistically significant. For the liver, the overall mean difference between the hybrid IR and DLR was 0.633 HU (*P* = .483, SD ± 0.902 HU).^[3]^ In a study by Kaga et al,^[9]^ a statistically significant difference of only 3.1 HU was found in the CT density of the liver during the portal phase between the 2 reconstruction algorithms. This trend was also observed in unenhanced liver parenchyma,^[29]^ although non-significant, with a mean HU value of 58.6 HU and 60.3 HU for IR and DLR, respectively (*P* = .45) in contrast to other abdominal organs.

In a study by Kim et al,^[30]^ there was a trend of the mean CT values reconstructed by DLR being higher than those reconstructed by hybrid IR for the liver, with a mean difference of 5.2 HU, although the difference was not statistically significant.

In the study of Zhang et al^[31]^ which used the same vendor as our study, the mean CT values of the liver parenchyma on non-injected DLR was slightly superior to those of hybrid-IR (57.70 ± 8.36 vs 56.30 ± 8.87, respectively) but the difference was not statistically significant (*P* = .389). These results are consistent with our data, as all these studies showed a significantly increased SNR with DLR than with hybrid IR, in addition to better image quality and a lower radiation dose.

To our knowledge, there are no data in the literature on the effect of reduced iodine load on the liver parenchyma using a DLR algorithm with an intra-individual evaluation. Our results showed that the 90%IC of the liver attenuation value during the portal phase in group B (DLR) was 100.8 to 105.9, meaning that its value is superior to 100.8 in 95% of cases. Although this is slightly lower than the usual HU value for the liver parenchyma during the portal phase, the CNR of the liver/VP was not significantly different between the 2 groups. In contrast, Zeng et al study,^[28]^ which used a dedicated DLP algorithm on an Asian CT vendor, found a mean value of 109.9 UH for the liver parenchyma during the portal phase using a conventional contrast agent.

A recent study on coronary CTA^[32]^ with the same vendor as our study investigated the influence of 3 different image reconstructions (FBR, hybrid IR, and DLR) with different contrast media iodine concentrations (Iohexol 370, Iohexol-300, Iohexol 240). They found that using DLR with the lowest concentration (Iohexol 240) yielded comparable attenuation to hybrid IR with Iohexol 300, with a higher SNR, CNR, and image quality for DLR. This study used the same flow rate and tube voltage as our study.

Two types of vendor-specific DLR algorithms are clinically available for CT.^[33]^ The first commercialized DLR algorithm was the AiCE (Canon Medical Systems). This algorithm was trained on simulated low-dose HIR (hybrid IR) data and high-dose MBIR (Model-Based IR) data, which were used as the training input and training target, respectively, with multiple iterations. In clinical practice, the trained DLR engine is applied to hybrid IR images to generate high-quality AiCE images with multiple modes specialized for different anatomic areas. The second commercially available DLR algorithm is TrueFidelity (GE Healthcare), which uses higher-dose FBP images as target data, based on the concept of replicating the noise texture and visual impression of the FBP images. Unlike the AiCE algorithm, the TrueFidelity engine produces DLR images directly from projection data. Most papers in the literature that study the correlation between DLR and hybrid IR on liver parenchyma have used the GE algorithm. Only 2 papers have used the same vendor as the one we used (Canon Medical Systems).^[31,32]^ A possible explanation for this apparent disagreement with the results between all published papers could be the type of vendor used, with its specific DLR algorithm. In the DLR, the quality of the training target determines the performance of the output. Consequently, more research is needed to determine whether the different conceptions of the DLR algorithm could modify certain results in the final image.

Therefore, we suggest that using DLR with a low iodine concentration can provide sufficient enhancement of the liver parenchyma, in addition to other improved image parameters and a low radiation dose, with no inferiority compared to conventional contrast agents.

However, the current study has several limitations.

First, the study population was relatively small, but the confrontation was intraindividual and in the same range as most other published studies. Our investigation was retrospective and was performed at a single institution. Therefore, our findings must be considered preliminary results.

Second, this study focused only on the liver parenchyma because it is the largest organ with a homogeneous pattern in the portal phase, in contrast to the kidney parenchyma.

Third, because we considered this work as preliminary, we did not evaluate anomalies of the liver parenchyma. This study does not contain any information on the detection or characterization of hepatic lesions, which should be explored in a complementary study.

## Author contributions

**Conceptualization:** Sebastien Moliére, Catherine Roy.

**Data curation:** Gaspard Ludes.

**Formal analysis:** Mickael Ohana, Aissam Labani, Nicolas Meyer, Catherine Roy.

**Investigation:** Gaspard Ludes, Sebastien Moliére, Catherine Roy.

**Methodology:** Mickael Ohana, Aissam Labani, Sebastien Moliére, Catherine Roy.

**Resources:** Aissam Labani, Sebastien Moliére.

**Supervision:** Gaspard Ludes, Mickael Ohana, Sebastien Moliére, Catherine Roy.

**Validation:** Mickael Ohana, Sebastien Moliére, Catherine Roy.

**Visualization:** Aissam Labani, Catherine Roy.

**Writing – original draft:** Sebastien Moliére.

**Writing – review & editing:** Catherine Roy.

## References

[1] Brenner DJ Hall EJ. Computed tomography: an increasing source of radiation exposure. N Engl J Med. 2007;357:2277–84. 18046031 10.1056/NEJMra072149

[2] Geyer LL Schoepf UJ Meinel FG. State of the art: iterative CT reconstruction techniques. Radiology. 2015;276:339–57. 26203706 10.1148/radiol.2015132766

[3] Van Stiphout JA Driessen J Koetzier LR. The effect of deep learning reconstruction on abdominal CT densitometry and image quality: a systematic review and meta-analysis. Eur Radiol. 2022;32:2921–9. 34913104 10.1007/s00330-021-08438-z PMC9038933

[4] Arndt C Güttler F Heinrich A. Deep learning CT image reconstruction in clinical practice. Fortschr Röntgenstr. 2021;193:252–61. 10.1055/a-1248-2556 33302311

[5] Bernard A Comby PO Lemogne B. Deep learning reconstruction versus iterative reconstruction for cardiac CT angiography in a stroke imaging protocol: reduced radiation dose and improved image quality. Quant Imaging Med Surg. 2021;11:392–401. 33392038 10.21037/qims-20-626 PMC7719916

[6] Singh R Digumarthy SR Muse VV. Image quality and lesion detection on deep learning reconstruction and iterative reconstruction of sub millisievert chest and abdominal CT. Am J Roentgenol. 2020;214:566–73. 31967501 10.2214/AJR.19.21809

[7] Willemink MJ Noel PB. The evolution of image reconstruction for CT-from filtered back projection to artificial intelligence. Eur Radiol. 2019;29:2185–95. 30377791 10.1007/s00330-018-5810-7 PMC6443602

[8] Jensen CT Liu X Tamm EP. Image quality assessment of abdominal CT by use of new deep learning image reconstruction: initial experience. Am J Roentgenol. 2020;215:50–7. 32286872 10.2214/AJR.19.22332

[9] Kaga T Noda Y Fujimoto K. Deep-learning-based image reconstruction in dynamic contrast-enhanced abdominal CT: image quality and lesion detection among reconstruction strength levels. Clin Radiol. 2021;76:710.e15–24. 10.1016/j.crad.2021.03.010 33879322

[10] Nakamura Y Narita K Higaki T. Diagnostic value of deep learning reconstruction for radiation dose reduction at abdominal ultra-high-resolution CT. Eur Radiol. 2021;31:4700–9. 33389036 10.1007/s00330-020-07566-2

[11] Noda Y Iritani Y Kawai N. Deep learning image reconstruction for pancreatic low-dose computed tomography: comparison with hybrid iterative reconstruction. Abdom Radiol (NY). 2021;46:4238–44. 33973060 10.1007/s00261-021-03111-x

[12] Park C Choo KS Jung Y. CT iterative vs deep learning reconstruction: comparison of noise and sharpness. Eur Radiol. 2021;31:3156–64. 33057781 10.1007/s00330-020-07358-8

[13] Yoo YJ Choi IY Yeom SK. Evaluation of abdominal CT obtained using a deep learning based image reconstruction engine compared with CT using adaptive statistical iterative reconstruction. J Belgian Soc Radiol. 2022;106:15–8. 10.5334/jbsr.2638 PMC8992765 35480337

[14] Akagi M Nakamura Y Higaki T. Deep learning reconstruction improves image quality of abdominal ultra-high-resolution CT. Eur Radiol. 2019;29:4526–7. 31134364 10.1007/s00330-019-06249-x

[15] Behrendt FF Mahnken AH Keil S. Contrast enhancement in multidetector-row computed tomography (MDCT) of the abdomen: intraindividual comparison of contrast media containing 300 mg versus 370 mg iodine per ml. Eur Radiol. 2008;18:1199–205. 18228023 10.1007/s00330-008-0861-9

[16] Furuta A Ito K Fujita T. Hepatic enhancement in multiphasic contrast-enhanced MDCT: comparison of high- and low-iodine-concentration contrast medium in same patients with chronic liver disease. Am J Roentgenol. 2004;183:157–62. 15208131 10.2214/ajr.183.1.1830157

[17] Suzuki H Oshima H Shiraki N. Comparison of two contrast materials with different iodine concentrations in enhancing the density of the aorta, portal vein and liver at multi-detector row CT: a randomized study. Eur Radiol. 2004;14:2099–104. 15309493 10.1007/s00330-004-2439-5

[18] Tsurusaki M Sugimoto K Fuji M. Multi-detector row helical CT of the liver: quantitative assessment of iodine concentration of intravenous contrast material on multiphasic CT-a prospective randomized study. Radiat Med. 2004;22:239–45. 15468944

[19] Botsikas D Barnaure I Terraz S. Value of liver computed tomography with iodixanol 270, 80 kVp and iterative reconstruction. World J Radiol. 2016;8:693–9. 27551339 10.4329/wjr.v8.i7.693 PMC4965353

[20] Ichikawa S Motosugi U Shimizu T. Diagnostic performance and image quality of low-tube voltage and low-contrast medium dose protocol with hybrid iterative reconstruction for hepatic dynamic CT. Br J Radiol. 2021;94:20210601. 10.1259/bjr.20210601 PMC8631019 34586900

[21] Taguchi N Oda S Utsunomiya D. Using 80 kVp on a 320-row scanner for hepatic multiphasic CT reduces the contrast dose by 50 % in patients at risk for contrast-induced nephropathy. Eur Radiol. 2017;27:812–20. 27240454 10.1007/s00330-016-4435-y

[22] Choi MH Lee YJ Jung SE. The image quality and diagnostic performance of CT with low-concentration iodine contrast (240 mg Iodine/mL) for the abdominal organs. Diagnostics. 2022;12:752. 35328304 10.3390/diagnostics12030752 PMC8947528

[23] Hwang I Cho JY Kim SY. Low tube voltage computed tomography urography using low-concentration contrast media: comparison of image quality in conventional computed tomography urography. Eur J Radiol. 2015;84:2454–63. 26388465 10.1016/j.ejrad.2015.09.010

[24] Zhang H Ma Y Lyu J. Low kV and Low Concentration contrast agent with iterative reconstruction of computed tomography (CT) coronary angiography: a preliminary study. Med Sci Monit. 2017;23:5005–10. 29051477 10.12659/MSM.904251 PMC5661743

[25] Cao L Liu X Li J. A study of using a deep learning image reconstruction to improve the image quality of extremely low-dose contrast-enhanced abdominal CT for patients with hepatic lesions. Br J Radiol. 2021;94:20201086. 10.1259/bjr.20201086 PMC7934287 33242256

[26] Li LL Wang H Song J. A feasibility study of realizing low-dose abdominal CT using deep learning image reconstruction algorithm. J X-Ray Sci Technol. 2021;29:361–72. 10.3233/XST-200826 33612538

[27] Njølstad T Schulz A Godt JC. Improved image quality in abdominal computed tomography reconstructed with a novel Deep Learning Image Reconstruction technique – initial clinical experience. Acta Radiologica Open. 2021;10:1–9. 10.1177/20584601211008391 PMC8040588 33889427

[28] Zeng L Xu X Zeng W. Deep learning trained algorithm maintains the quality of half-dose contrast-enhanced liver computed tomography images: comparison with hybrid iterative reconstruction Study for the application of deep learning noise reduction technology in low dose. Eur J Radiol. 2021;135:109487. 33418383 10.1016/j.ejrad.2020.109487

[29] Kaga T Noda Y Mori T. Unenhanced abdominal low-dose CT reconstructed with deep learning-based image reconstruction: image quality and anatomical structure depiction. Jpn J Radiol. 2022;40:703–11. 35286578 10.1007/s11604-022-01259-0 PMC9252942

[30] Kim JH Yoon HJ Lee E. Validation of deep-learning image reconstruction for low-dose chest computed tomography scan: emphasis on image quality and noise. Korean J Radiol. 2021;22:131–8. 32729277 10.3348/kjr.2020.0116 PMC7772377

[31] Zhang X Zhang G Xu L. Application of deep learning reconstruction of ultra-low-dose abdominal CT in the diagnosis of renal calculi. Insights Imaging. 2022;13:163. 36209195 10.1186/s13244-022-01300-w PMC9547757

[32] Otgonbaatar C Ryu J-K Shin J. Deep learning reconstruction allows for usage of contrast agent of lower concentration for coronary CTA than filtered back projection and hybrid iterative reconstruction. Acta Radiol. 2022;64:1007–17. 35979586 10.1177/02841851221118476

[33] Nagayama Y Sakabe D Goto M. Deep learning–based reconstruction for lower-dose pediatric CT: technical principles, image characteristics, and clinical implementations. Radiographics. 2021;41:1936–53. 34597178 10.1148/rg.2021210105

